# The Protective Effect of Boric Acid Against High Fructose-Induced Liver and Kidney Damage in Rats

**DOI:** 10.1007/s12011-025-04542-z

**Published:** 2025-02-06

**Authors:** Duygu Yüksel, Mehmet Başeğmez, Fahriye Kan

**Affiliations:** 1 Department of Medical Services and Techniques, Vocational School of Health Services, Gümüşhane University, Pathology Program, Gümüşhane, Turkey; 2 Department of Veterinary, Laboratory and Veterinary Health Program, Acıpayam Vocational High School, Pamukkale University, Denizli, Turkey; 3 Department of Biochemistry, Faculty of Veterinary Medicine, Afyon Kocatepe University, Afyonkarahisar, Turkey

**Keywords:** Boric acid, High fructose, Kidney, Liver, Rat

## Abstract

This study aimed to determine the protective role of boric acid (BA) in high fructose (HF)-induced liver and kidney toxicity in a young rat model. High-fructose consumption causes serious damage to liver and kidney tissue in healthy individuals and contributes to the emergence of various metabolic diseases. Thirty-two healthy female Wistar albino rats (250–300 g weight and 3–4 months) were randomly distributed into four equal groups (*n* = 8): control, high fructose % 20 (HF), boric acid 20 mg/kg (BA), and HF + BA. High fructose was freshly prepared and administered to the rats as 20 g of d-fructose dissolved in 100 mL of tap water daily for a duration of 30 days. Boric acid (20 mg/kg) was administered through gastric gavage throughout the 30-day study period. At the end of study, blood, liver, and kidney were collected from rats. The results indicated that high fructose induced increased glucose, total cholesterol, triglyceride, and urea levels in rat serum. Boric acid administration significantly decreased glucose, total cholesterol, triglyceride, and urea levels in HF + BA groups. The results indicated that high fructose-induced oxidative stress by increasing the level of MDA and by decreasing GSH levels, and CAT activity in the liver and kidney of rats. However, oral BA administration significantly decreased MDA levels and increased GSH levels, and CAT activity (*p* < 0.05). Furthermore, BA significantly reduced high fructose-induced histopathological and Immunohistochemistry alteration in the liver and kidney tissues. In conclusion, BA may prevent the oxidative imbalance and histopathological and immunohistochemical damage caused by high fructose in liver and kidney tissues in rats.

## Introduction

Fructose, which occurs naturally in fruits, honey, and some vegetables, is usually taken into the body as a nutrient from industrial and commercial products such as sweetened soft drinks and high fructose corn syrup (HFCS) [1]. Fructose, which is used as an important nutrient in diets, is increasingly used today [2]. Muriel et al. [3] report a significant increase in the adoption of fructose-enriched diets worldwide. High fructose (HF) consumption leads to chronic conditions like metabolic syndrome (MetS), contributing to the emergence of several metabolic illnesses, including obesity, dyslipidemia, and hypertension in otherwise healthy individuals [4]. Zhang et al. [5] have shown that high-fructose intake causes oxidative stress and liver damage by producing inflammatory cytokines, adiponectin, leptin, and endotoxins. Indeed, Li et al. [6] and Saleh et al. [7] reported that fructose induced very early histological changes, such as increased glomerular cell proliferation and infiltration of mononuclear cells, as well as functional changes such as glomerular hyperfiltration and sodium retention in rat kidneys.

The production of reactive oxygen species (ROS) due to oxidative stress is associated with various disease disorders, including apoptosis, cellular proliferation, and organ malfunction [8]. Iskender et al. [9] reported that fructose caused oxidative damage by increasing malondialdehyde (MDA) levels, an indicator of lipid peroxidation, in the liver tissues of rats. Furthermore, Niazi et al. [10] demonstrated that fructose consumption damages rats’ livers by enhancing oxidative stress and lipid peroxidation.

Boric acid, commonly used in industrial applications, demonstrated beneficial effects for both humans and animals in several experimental investigations [11]. In addition, a recent study found that a variety of medical fields use boron-containing medications [12]. Boron compounds are generally available as boric acid and disodium tetraborate (borax) [13]. A necessary element, boron serves as a cofactor for several enzymes involved in cell division and the metabolism of the majority of proteins, carbs, and fats [14].

The aim of this study was to investigate the biochemical, histopathological, and immunohistochemical effects of HF consumption on liver and kidney tissues in young rats.

## Materials and Methods

### Chemical

In this present study, a hepatoxicity and nephrotoxicity model in rats was constructed using 20% d-fructose solution (Biomatic 99%, CAS: 57–48-7, MW: 180.16). Boric acid (H3BO3) (Code number: V55901), purchased from Chemistry Lab Istanbul, Türkiye, was used as a test compound. Fructose-enriched beverages were prepared fresh daily. To make a 20% fructose solution daily, 20 g of d-fructose were dissolved in 100 mL of tap water. All the other chemicals and reagents were of analytical reagent grade purchased from commercial sources.

### Animals

The Pamukkale University Animal Experiments Ethics Committee approved the commencement of this study (study number PAUHDEK-2023/35). Thirty-two Wistar-Albino female rats (250–300 g and 3–4 months) were obtained from Pamukkale University Experimental Surgery Application and Research Center, housed under environmental conditions (22 ± 1 °C), humidity (50 ± 5%), with 12 h light–dark cycle and provided with standard pelleted rat diet. The rats obtained daily examinations under veterinary supervision throughout the study. A standard commercial chow diet (Korkuteli Yem Gıda San. A.Ş., Antalya, Turkey) and fresh drinking water were made available ad libitum to all rats throughout the study period. The experiment adhered to the ARRIVE 2.0 guidelines.

### Experimental Design

Rats were divided into four experimental groups, each containing eight rats (*n* = 8); control, high fructose % 20 (HF), boric acid 20 mg/kg (BA), and HF + BA. The control group rats were given standard rat chow and fresh drinking water. The rats in the high-fructose group were given 20 g of d-fructose dissolved in 100 mL of tap water, freshly prepared daily [7]. The rats in the boric acid group were administered BA 20 mg/kg orally via gastric gavage throughout the study period [15]. The rats in the HF + BA group were given 20 mg/kg of BA orally along with 20 g of d-fructose dissolved in 100 mL of tap water. HF and BA were administered over the 30-day study period. At the end of the study, blood, liver, and kidney tissues were collected from rats anesthetized with ketamine (87 mg/kg) and xylazine (13 mg/kg). Serum samples taken for biochemical analysis were stored in a deep freezer at − 80 °C until the analysis stage. Liver and kidney tissues were removed. Some of these tissues were fixed in formalin at a concentration of 10% for histopathological and immunohistochemical examination analysis. From the other part of the tissues, supernatants were obtained and stored at − 80 °C until the analysis stage.

### Biochemical Analyses

Blood samples taken into serum biochemistry tubes were centrifuged at 3000 rpm at + 4 °C for 15 min, and serum samples were obtained. Serum aspartate amino transferase (AST), alanine aminotransferase (ALT), alkaline phosphatase (ALP) activities, protein, urea, creatine, cholesterol, triglyceride (HUMAN, Wiesbaden, Germany), and glucose (BIOLABO, Maizy, France) levels were measured spectrophotometrically using commercial test kits (Thermo Fisher Scientific Oy Ratastie 2, FI-01620 Vantaa, Finland).

The liver and kidney tissues were washed thoroughly with cold 0.9% sodium chloride (NaCl). Then they were homogenized in 0.1 M phosphate buffer (pH = 7.4) at a ratio of 1:40 w/v. The tissue homogenates were centrifuged at 5000 rpm for 15 min using the method described by Küçükkurt et al. [16]. Subsequently, the concentrations of MDA, glutathione (GSH), superoxide dismutase (SOD), and catalase (CAT) were measured from the supernatants of the obtained liver and kidney tissues.

Lipid peroxidation was assayed by measuring the level of MDA in liver and kidney tissue supernatant. The MDA levels were determined using the method described by Ohkawa et al. [17] and recorded as nmol/mL. The GSH concentration of tissue supernatant was measured using the method described by Beutler et al. [18] and recorded as nmol/mL. The superoxide dismutase activity of tissue supernatant was measured using the method of Sun et al. [19] and recorded as U/mgHb. The catalase enzyme activity of tissue supernatant was measured using the Aebi [20] method and recorded as U/mgHb.

### Histopathological Analyses

Liver and kidney tissue samples were subjected to 10% formaldehyde fixation. Following fixation in paraffin wax, liver and kidney tissue samples were carefully processed with a fully automated tissue processor (Leica ASP300S, Leica Microsystems, Nussloch, Germany). After 4 to 5 h of cooling, the tissue samples were sectioned at 5 μm thickness using a rotary microtome (Leica 2155, Leica Microsystems, Nussloch, Germany). Later, sections were processed for hematoxylin–eosin (HE) coloring, and for the examination of the samples, a light microscope was utilized.

### Immunohistochemistry

The sections were stained using the streptavidin–biotin complex peroxidase technique in accordance with the manufacturer’s instructions. Primary antibodies for inducible nitric oxide synthase (iNOS) [Anti-iNOS antibody (ab15323)] and hypoxia-inducible factor-1α (HIF-1α) [Anti-HIF-1 alpha antibody—C-terminal (ab228649)] were used in 1/100 dilution for this purpose. The secondary kit was the EXPOSE Mouse and Rabbit Specific HRP/DAB Detection IHC kit (ab80436) and the chromogen was 3,3-diaminobenzidine (DAB) (Abcam, Cambridge, UK), and Harris hematoxylin was then used as a counterstain. The Database Manual Cell Sens Life Science Imaging Software System (Olympus Corporation, Tokyo, Japan) was used for microphotography and morphometric analysis. All slides were examined for immunopositivity.

The pathological analyses were independently evaluated by a pathologist from another university through a blind review process.

### Statistical Analysis

Statistical analysis was carried out using the SPSS 25.0 statistical package (IBM SPSS Statistics, IL, USA). Data are presented as mean ± standard deviation (SD) with 95% confidence interval. The GraphPad Prism 10 (GraphPad Software, San Diego, CA, USA) was used for graphical illustrations. Shapiro–Wilk and Levene’s tests were applied to evaluate the data normality and homogeneity, respectively. One-way analysis of variance (ANOVA) was used to compare the means of the multiple groups, and Duncan’s post hoc comparison tests were then performed. The level of significance was considered *p* < 0.05.

## Results

### Biochemical Evaluation

**Fig. 1:**
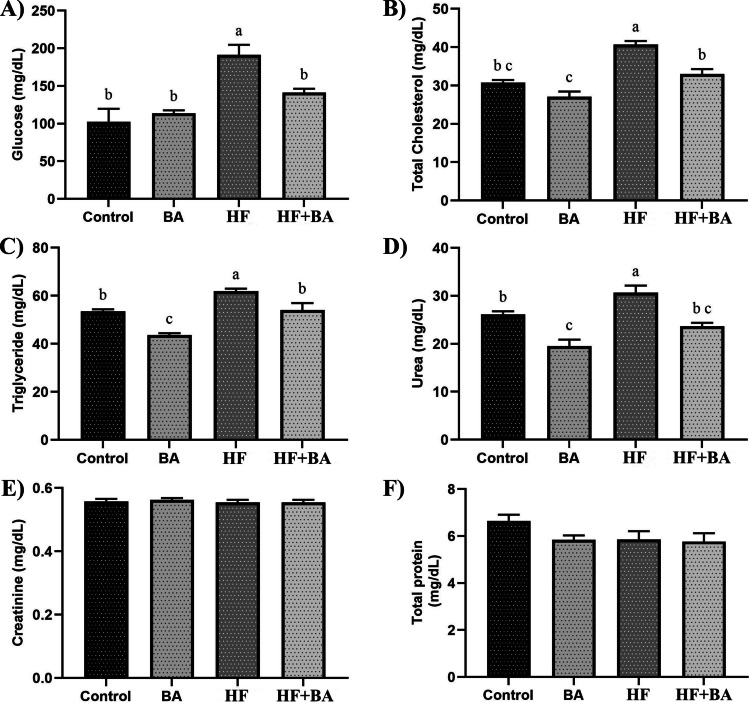
Effects of boric acid (BA) on serum glucose, total cholesterol, triglyceride, urea, creatinine, and total protein levels in high fructose (HF)-induced rats. The values were expressed as means ± SD. The different superscripts a, b, and c showed statistically significant differences within the same column (*p* < 0.05). The same superscripts a, b, and c did not show statistically significant differences within the same column (*p* > 0.05)

As shown in Fig. 1, HF exposure significantly increased glucose, total cholesterol, triglyceride, and urea levels compared to control, BA, and HF + BA groups in rat serum (*p* < 0.05). BA administration significantly decreased glucose, total cholesterol, triglyceride, and urea levels in HF + BA groups (*p* < 0.05). There is no significant difference between all groups for creatinine, and total protein levels (*p* > 0.05).

**Fig. 2:**
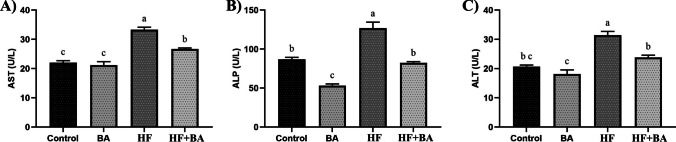
Effects of boric acid (BA) on serum aspartate aminotransferase (AST), alkaline phosphatase (ALP), and alanine aminotransferase (ALT) levels in high fructose (HF)-induced rats. The values were expressed as means ± SD. The different superscripts a, b, and c showed statistically significant differences within the same column (*p* < 0.05). The same superscripts a, b, and c did not show statistically significant differences within the same column (*p* > 0.05)

As shown in Fig. 2, HF exposure significantly increased AST, ALP, and ALT levels compared to control, BA, and HF + BA groups in rat serum (*p* < 0.05). BA administration significantly decreased AST, ALP, and ALT levels in HF + BA groups (*p* < 0.05).

**Fig. 3:**
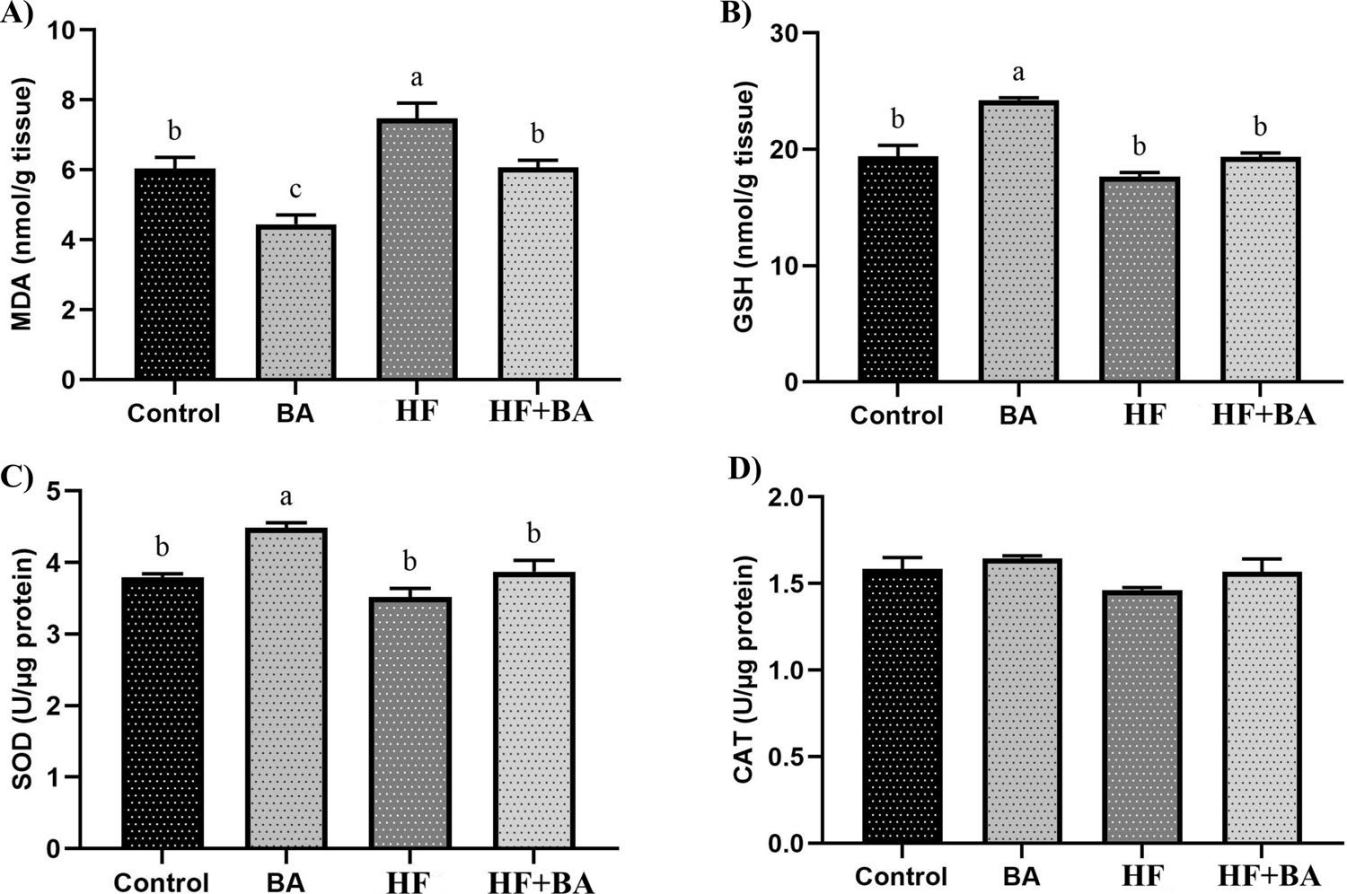
Effects of boric acid (BA) on malondialdehyde (MDA), reduced glutathione (GSH), superoxide dismutase (SOD), and catalase (CAT) levels in high fructose (HF)-induced rat liver. The values were expressed as means ± SD. The different superscripts a, b, and c showed statistically significant differences within the same column (*p* < 0.05). The same superscripts a, b, and c did not show statistically significant differences within the same column (*p* > 0.05)

As shown in Fig. 3A, high-fructose exposure significantly increased the MDA level in the liver compared to the control group (*p* < 0.05). BA co-administration decreased MDA levels in the HF + BA group (*p* < 0.05). In addition, BA treatment alone decreased MDA levels compared to the control group (*p* < 0.05). As shown in Fig. 3B, HF exposure did not alter GSH levels to statistical significance liver compared to the control group (*p* > 0.05). Additionally, BA co-administration did not change GSH levels in the HF + BA group (*p* > 0.05). However, BA treatment alone significantly increased GSH levels compared to the control group (*p* < 0.05). As shown in Fig. 3C, HF exposure did not alter SOD levels to statistical significance liver compared to the control group (*p* > 0.05). Additionally, BA co-administration did not change SOD levels in the HF + BA group (*p* > 0.05). However, BA treatment alone significantly increased SOD levels compared to the control group (*p* < 0.05). As shown in Fig. 3D, there is no significant difference between all groups for CAT levels (*p* > 0.05).

**Fig. 4:**
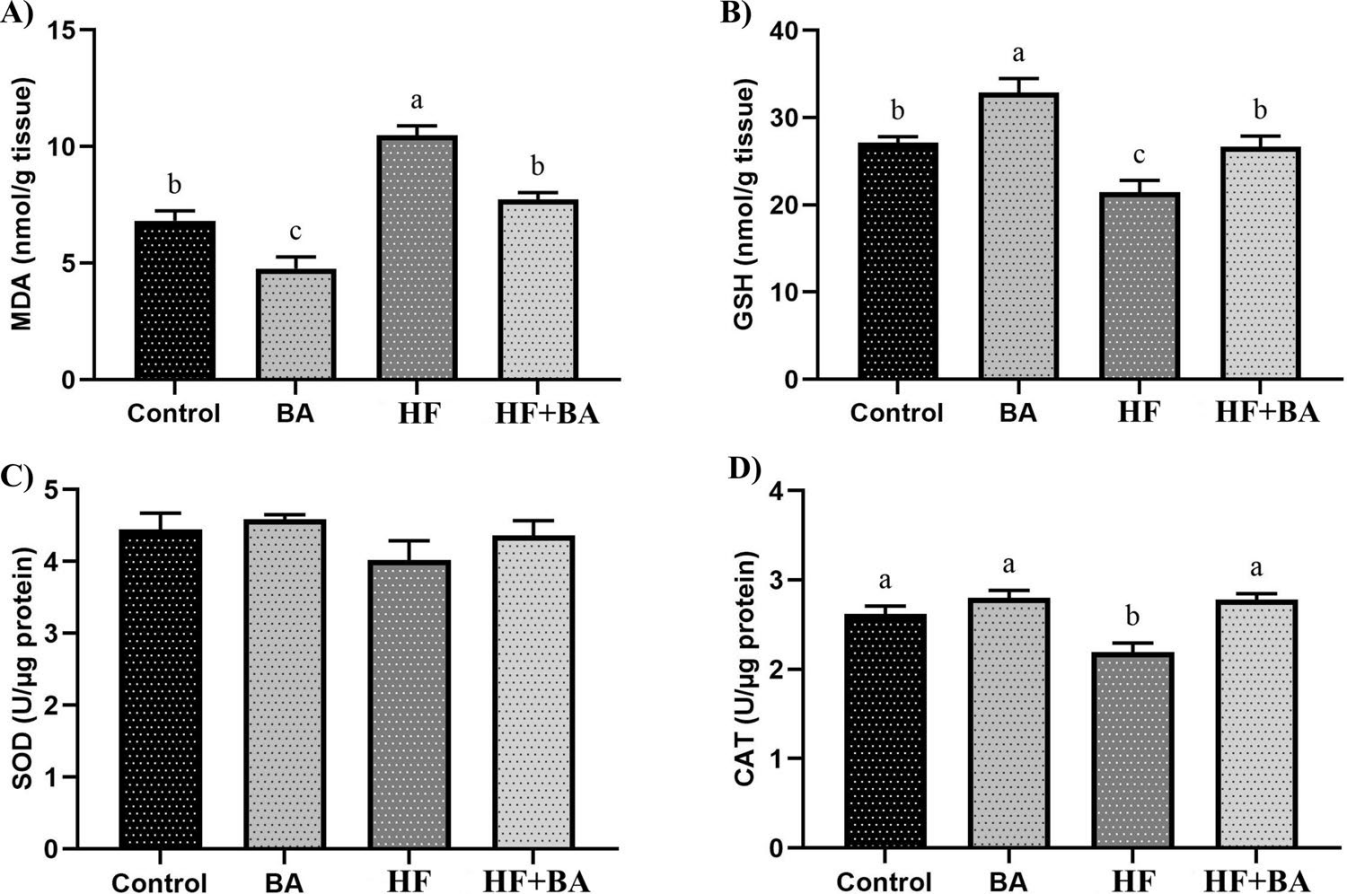
Effects of boric acid (BA) on malondialdehyde (MDA), reduced glutathione (GSH), superoxide dismutase (SOD), and catalase (CAT) levels in high fructose (HF)-induced rat kidney. The values were expressed as means ± SD. The different superscripts a, b, and c showed statistically significant differences within the same column (*p* < 0.05). The same superscripts a, b, and c did not show statistically significant differences within the same column (*p* > 0.05)

As shown in Fig. 4A, high-fructose exposure significantly increased the MDA level in the kidney compared to the control group (*p* < 0.05). BA co-administration decreased MDA levels in the HF + BA group (*p* < 0.05). In addition, BA treatment alone decreased MDA levels in the kidney compared to the control group (*p* < 0.05). As shown in Fig. 4B, HF exposure significantly decreased GSH levels in the kidney compared to the control group (*p* < 0.05). Co-administration with BA increased kidney GSH levels in HF + BA group (*p* < 0.05). Further, BA treatment alone significantly increased GSH levels in the kidney compared to the control group (*p* < 0.05). As shown in Fig. 4C, there is no significant difference between all groups for SOD levels (*p* > 0.05). As shown in Fig. 4D, CAT levels were decreased in the HF group compared to all groups (*p* < 0.05). Co-administration with BA increased kidney CAT levels in the HF + BA group (*p* < 0.05).

### Histopathological Evaluation

**Fig. 5:**
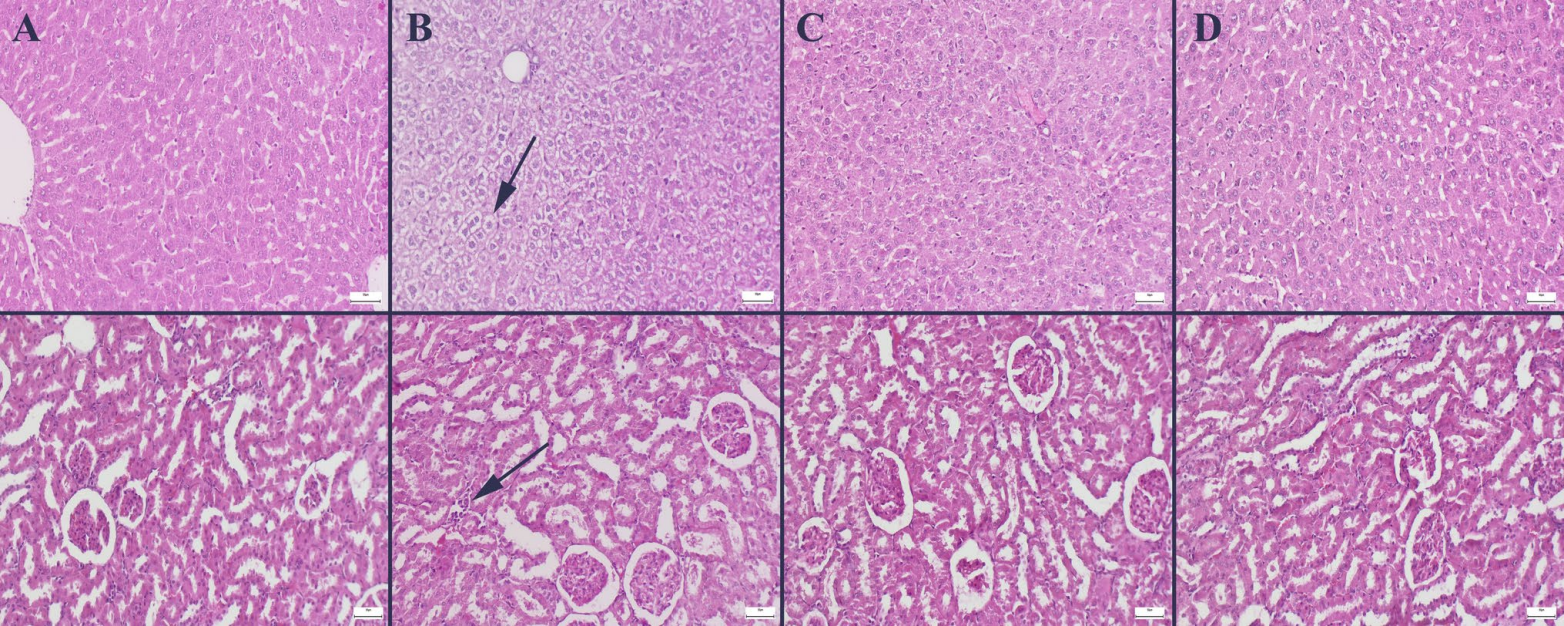
Representative histopathological findings of liver (upper row) and kidney (below row) between the group. **A** Normal tissue architecture in control group, **B** marked lipidosis in liver and inflammatory cell infiltrations in HF group, **C** marked amelioration in HF + BA, **D** normal tissue histology in BA group, HE, bars = 50 μm

The results of this study reveal that adding HF to the diet of young rats leads to pathological findings in the liver and kidneys, but BA shows a prophylactic effect in improving these findings. The liver and renal tissues of the control and boric acid groups showed no abnormal findings at the time of the histological evaluation. There were reports of severe lipidosis, hyperemia, and hemorrhages in hepatocytes from the HF groups. Significant hyperemia, hemorrhagic patches, and infiltrations of inflammatory cells were observed in the kidney of the HF group. In the HF + BA group, boron treatment improved the liver and kidney results (Fig. 5).

### Immunohistochemical Evaluation

**Fig. 6:**
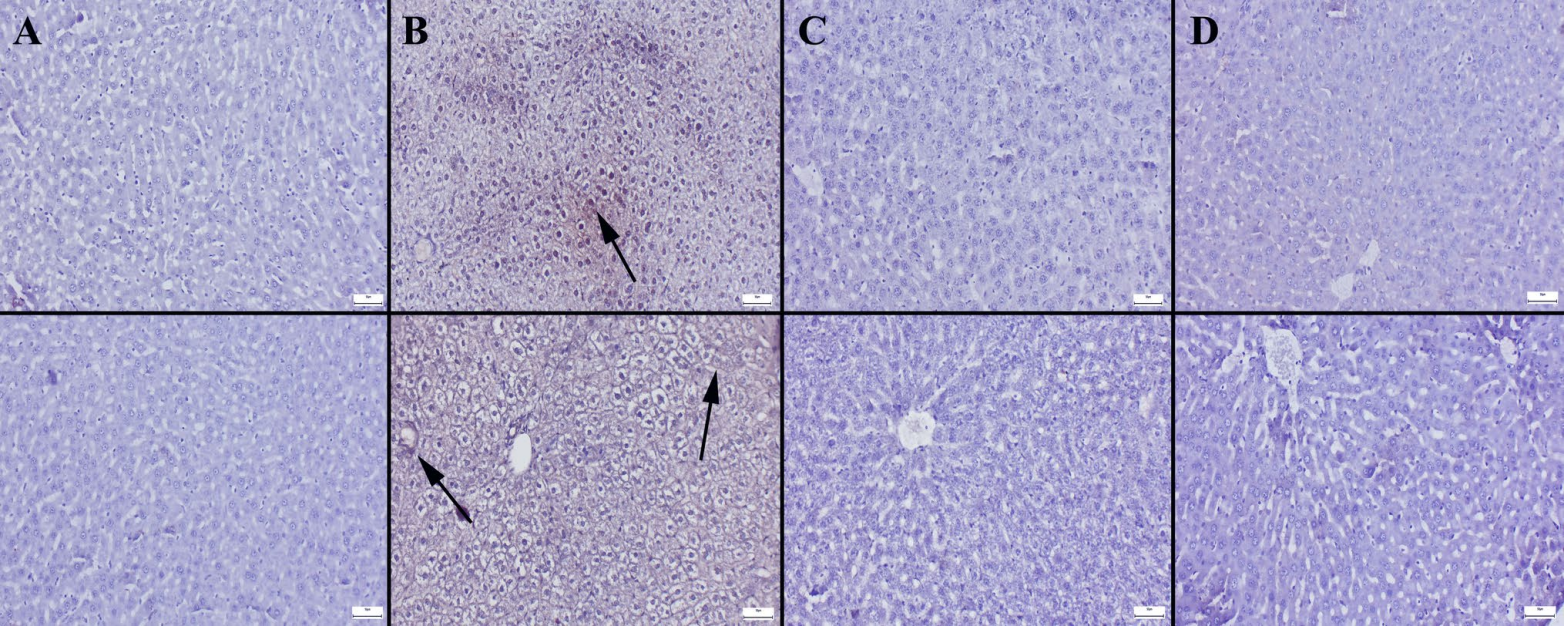
Immunohistochemical iNOS (upper row) and HIF-1α (below row) expressions of liver between the groups. **A** Negative expressions in control group, **B** moderate expressions (arrows) in HF groups, **C** decreased expressions in HF + BA group, **D** no expression in BA group, streptavidin biotin method, scale bars = 50 μm

**Fig. 7:**
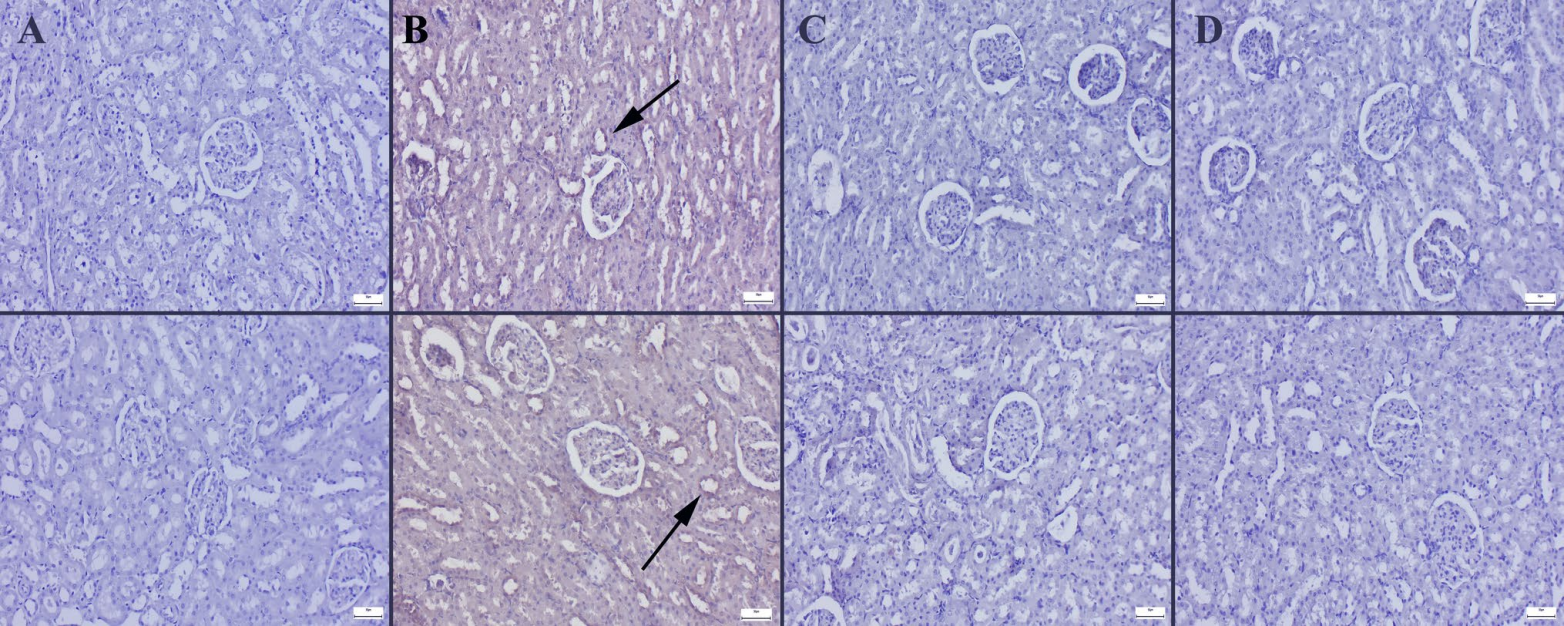
Immunohistochemical iNOS (upper row) and HIF-1α (below row) expressions of kidneys between the groups. **A** No expressions in control group, **B** marked expressions (arrows) in HF groups, **C** decreased expressions in HF + BA group, **D** negative expression in BA group, streptavidin biotin method, scale bars = 50 μm

Animals fed HF showed increased expressions of iNOS and HIF-1α in their liver and kidneys. The severe lipidosis in the HF group caused the manifestations in the liver to be mainly mild. In the kidneys, tubular epithelial cells frequently displayed the markers for the HF group. Treatment with BA reduced the expression levels of the kidneys and liver (Figs. 6, 7).

Data were presented as mean ± SD (*n* = 8). The different superscripts, a and b, showed statistically significant differences within the same row (*p* < 0.05). The same superscripts, a and b, did not show statistically significant differences within the same row (*p* > 0.05). Abbreviations: *İNOS*, İnducible nitric oxide synthase; *HIF-1α*, hypoxia-inducible factor 1-alpha; *SD*, standard deviation.

## Discussion

High fructose, one of the industrial and commercial products with a high volume of distribution, continues to pose a danger to human and animal health today [21]. Fructose enters the redox cycle and depletes GSH levels, SOD, and CAT antioxidant enzyme activities, resulting in increased production of reactive oxygen species (ROS) [22, 23]. Excessive consumption of a HF may lead to chronic liver [24] and kidney [25] damage due to increased lipid peroxidation. Furthermore, studies have shown that a fructose-enriched diet has detrimental histopathological and immunohistochemical effects on liver [26] and kidney [22] tissue, but it also has protective effects of boron and boron compounds on liver, kidney [27], and biochemical parameters [15] (Table 1).

**Table 1:** Effects of boric acid (BA) on liver and kidney iNOS and HIF-1α immunohistochemical markers in high fructose (HF)-induced rats

Tissues	Groups	iNOS	HIF-1α
**Liver**	Control	0.14 ± 0.14^a^	0.14 ± 0.14^a^
	HF	1.57 ± 0.53^b^	1.28 ± 0.48^b^
	HF + BA	0.42 ± 0.20^a^	0.28 ± 0.18^a^
	BA	0.14 ± 0.14^a^	0.14 ± 0.14^a^
**Kidney**	Control	0.14 ± 0.14^a^	0.14 ± 0.14^a^
	HF	0.71 ± 0.18^b^	1.14 ± 0.69^b^
	HF + BA	0.14 ± 0.14^a^	0.28 ± 0.08^a^
	BA	0.14 ± 0.14^a^	0.14 ± 0.14^a^

Boron has been reported to form complexes with glycoproteins, glycolipids, and other hydroxyl-containing molecules, thus playing a critical role in the synthesis and metabolism of various biochemical reactions [28, 29]. In our recent study, we demonstrated that boric acid exerts dose-dependent protective effects on liver and kidney tissues in aged rats by modulating inflammation and oxidative imbalance [15]. In this study, rats were administered oral BA (20 mg/kg) and 20 g of d-fructose (HF) dissolved in 100 mL of tap water for 30 days, and serum biochemical parameters, oxidative indicators in the liver and kidneys, along with histological and immunohistochemical alterations were examined.

Oxidative stress is associated with a number of pathological conditions that cause tissue and organ dysfunction, and BA has ameliorative effects on this damage [15]. High-fructose consumption causes an increase in MDA [23, 30] and a decrease in antioxidant defense systems such as SOD, CAT, and GSH, [22, 23] leading to tissue and organ damage. The present study confirmed the detrimental effect of administering HF consumption on the antioxidative system in rats’ liver and kidney tissues. Compared to the control and BA groups, HF caused a significant increase in MDA levels in liver and kidney tissues, indicating a significant increase in lipid peroxidation. High-fructose consumption in the liver [31] and kidney [32] tissues of rats caused tissue damage by raising MDA levels, an indicator of lipid peroxidation. Similarly, our findings show that HF increases liver and kidney MDA levels while decreasing kidney GSH and CAT levels. In addition, similar to our study, rats given a fructose solution showed significant increases in total oxidant status levels in the liver, and SOD and CAT activities did not change [33]. However, the administration of BA demonstrated a healing effect against oxidative damage in kidney and liver tissue. As a matter of fact, this situation is in accordance with the study of Basegmez and Dogan [15], in which dose-dependent BA supplementation showed an ameliorative effect on liver and kidney tissue in rats.

Although the amount and type of boron produced different results in the glucose level, the general consensus is that boron tends to lower this level [34]. In a recent study, BA was shown to reduce high blood glucose levels in groups given streptozotocin [35]. Demirdogen [36] found a negative relationship between glucose level and serum boron level in diabetic and obese diabetic volunteers. In this present study, 20 mg/kg BA did not cause a significant difference in glucose level compared to the control group, but the increased glucose value in the HF group decreased in the HF + BA group. Indeed, the fact that boron-containing compounds raise the levels of genes linked to insulin production in pancreatic cells may explain this [37]. The increase in cholesterol and triglyceride levels of the HF diet in our study similarly supports the study by Saleh et al. [7]. We observed that BA, given in combination with a HF diet, reversed this increase. This is similar to the reported negative correlation between serum boron levels and total cholesterol and triglyceride levels [36]. These changes observed in cholesterol and triglyceride levels in the study indicate the positive effect of BA on lipid metabolism. Indeed, the report emphasized that boron may alter the lipid profile by affecting lipid metabolism, particularly triglyceride and very low-density lipoprotein (VLDL) secretion [38, 39]. In the present study, urea levels increased on a HF diet. BA administration decreased serum urea levels. Similarly, urea levels increased after poisoning and decreased after boron administration, and boron showed a protective effect [40, 41].

The liver, which has important functions in carbohydrate and lipid metabolism, is the organ most affected by metabolic changes and the first to be damaged [42]. Botezelli et al. [43] showed that HF administration in rats caused liver damage. Similarly, this present study demonstrated that the HF diet damaged the liver by increasing levels of the liver enzymes AST, ALP, and ALT. Additionally, this study supports Iskender et al. [42] and Yuan et al. [44] reports, which found that rats and mice fed an HF diet had higher serum AST, ALP, and ALT activities than the control group. Furthermore, the report that ALT and AST activities are higher in rats fed an HF diet compared to the control group indicates that the study is consistent [45, 46]. However, this study demonstrated that BA application had an improving effect on serum AST, ALP, and ALT liver enzyme levels. As a matter of fact, this situation supports the report that boron has a protective effect against hepatotoxicity [47]. However, this study is not consistent with a previous report [48] in which %10 HF consumption for 8 weeks had no effect on serum ALT and ALP levels in male rats. This discrepancy could be attributed to differences in the administered HF dose and the gender of the rats employed in the respective studies.

In addition, the study’s histopathological and immunohistochemical findings showed that HF consumption damaged the structure of liver and kidney tissues. Yuan et al. [44] showed that mice fed HF had more serious hepatocyte necrosis and cytoplasmic vacuolation in liver sections than mice in a control group [44]. Furthermore, the HF diet induced hemorrhage, inflammation, cytoplasmic vacuolation, necrosis, and apoptosis in the liver by triggering oxidative stress and inflammation in rats [30, 49]. Abdel-Kawi et al. [22] associated high-fructose intake with renal damage in rats, characterized by the shedding and dilatation of tubular epithelial cells in the cortex, along with the accumulation of interstitial collagen. Similarly, our findings showed that HF consumption caused severe lipidosis, hyperemia, and hemorrhages in the liver and significant hyperemia, hemorrhagic patches, and inflammatory cell infiltration in the kidney tissues, leading to histopathological damage. BA treatment reduced or eliminated these histopathological findings in rats exposed to HF consumption. On the other hand, in our study, the levels of HIF-1α and iNOS immunohistochemical staining were observed to be increased in the liver and kidney tissues of the HF groups compared to the other groups. HIF-1α is a transcription factor that regulates hypoxia and is essential for sustaining homeostasis in hypoxic conditions [50]. iNOS is an interesting marker that plays a key role in many physiological and pathophysiological conditions [51]. Aşcı et al. [50] demonstrate that 30% fructose syrup solution consumption increases HIF-1α expression immunohistochemically in cardiac tissues. Youssef [52] demonstrated a significant increase in iNOS expression in the centrilobular and periportal regions of liver sections from rats with high-fructose consumption. Similarly, our findings show that high-fructose consumption in rats leads to a significant increase in HIF-1α and iNOS immunoexpression in liver and kidney tissues. However, BA administration decreased HIF-1α and iNOS immunoexpression in HF consumption.

Our results conclusively show that BA has a lowering effect on HF-induced oxidative stress and histopathological damage. Consequently, BA may have an ameliorative effect on the liver and kidney tissue damage caused by HF.

## Conclusion

In this study, we found that of HF cause liver and kidney tissue damage in rats. Our findings indicated that HF-induced liver and kidney organ damage was associated with the induction of oxidative imbalances and biochemical parameters. We found that co-administration of BA decreased HF-induced liver and kidney organ damage. Our findings indicate that oral BA supplementation may exert a protective effect against oxidative stress-induced tissue damage in the liver and kidney caused by HF. In our future studies, we aim to investigate more detailed molecular mechanisms beyond the protective effects of boric acid on physiological systems.

## Data Availability

No datasets were generated or analysed during the current study.

## References

[1] Havel PJ (2005) Dietary fructose: implications for dysregulation of energy homeostasis and lipid/carbohydrate metabolism. Nutr Rev 63(5):133–157. 10.1111/j.1753-4887.2005.tb00132.x 15971409 10.1301/nr.2005.may.133-157

[2] Rippe JM, Angelopoulos TJ (2015) Fructose-containing sugars and cardiovascular disease. Adv Nutr 6(4):430–439. 10.3945/an.114.008177 26178027 10.3945/an.114.008177 PMC4496738

[3] Muriel P, López-Sánchez P, Ramos-Tovar E (2021) Fructose and the liver. Int J Mol Sci 22(13):69–69. 10.3390/ijms22136969 10.3390/ijms22136969 PMC8267750 34203484

[4] Kelishadi R, Mansourian M, Heidari-Beni M (2014) Association of fructose consumption and components of metabolic syndrome in human studies: a systematic review and meta-analysis. Nutrition 30(5):503–510. 10.1016/j.nut.2013.08.014 24698343 10.1016/j.nut.2013.08.014

[5] Zhang DM, Jiao RQ, Kong LD (2017) High dietary fructose: direct or indirect dangerous factors disturbing tissue and organ functions. Nutrients 9(4):335. 10.3390/nu9040335 28353649 10.3390/nu9040335 PMC5409674

[6] Li X, Zhang L, Yang Y, Wu D, Liu W, Li X, Su Z, Chen H, Wang L, Zheng L (2018) Histopathology and urinary metabonomics reveal the role of dietary salt on the pathogenesis of fructose-induced kidney injury. Int J Clin Exp Med 11(7):6662–6673

[7] Saleh R, Merghani BH, Awadin W (2017) Effect of high fructose administration on histopathology of kidney, heart and aorta of rats. Journal of Advanced Veterinary and Animal Research 4(1):71–79. 10.5455/javar.2017.d193

[8] Doğan MF, Kaya K, Demirel HH, Başeğmez M, Şahin Y, Çiftçi O (2023) The effect of vitamin C supplementation on favipiravir-induced oxidative stress and proinflammatory damage in livers and kidneys of rats. Immunopharmacol Immunotoxicol 45(5):521–526. 10.1080/08923973.2023.2181712 36794622 10.1080/08923973.2023.2181712

[9] Iskender H, Yenice G, TerimKapakin KA, Dokumacioglu E, Sevim C, Hayirli A, Altun S (2022) Effects of high fructose diet on lipid metabolism and the hepatic NF-κB / SIRT1 pathway. Biotech Histochem 97(1):30–38. 10.1080/10520295.2021.1890214 33629622 10.1080/10520295.2021.1890214

[10] Niazi AA, Gharaei FK, Saebinasab Z, Maleki M, Maghool F, Fereidooni F, Safari T (2021) Eugenol administration improves liver damage induced by a fructose-rich diet. Adv Biomed Res 10(1):42. 10.4103/abr.abr_237_20 35071110 10.4103/abr.abr_237_20 PMC8744418

[11] Uluisik I, Karakaya HC, Koc A (2018) The importance of boron in biological systems. J Trace Elem Med Biol 45:156–162. 10.1016/j.jtemb.2017.10.008 29173473 10.1016/j.jtemb.2017.10.008

[12] Soriano-Ursúa MA, Cordova-Chávez RI, Farfan-García ED, Kabalka G (2024) Boron-containing compounds as labels, drugs, and theranostic agents for diabetes and its complications. World J Diabetes 15(6):1060 38983826 10.4239/wjd.v15.i6.1060 PMC11229952

[13] Ince S, Erdogan M, Demirel HH, Agca Y, Dal G, Uguz C (2018) Boron enhances early embryonic gene expressions and improves fetal development of rats. J Trace Elem Med Biol 50:34–46. 10.1016/j.jtemb.2018.06.002 30262302 10.1016/j.jtemb.2018.06.002

[14] Prashanth L, Kattapagari KK, Chitturi RT, Baddam VRR, Prasad LK (2015) A review on role of essential trace elements in health and disease. J Dr YSR Univ Health Sci 4(2):75–85. 10.4103/2277-8632.158577

[15] Başeğmez M, Doğan MF (2024) Effects of boric acid on oxidant-antioxidant, proinflammatory cytokine levels, and biochemical parameters in aged rats. Pamukkale Medical Journal 17(2):369–379. 10.31362/patd.1438593

[16] Kucukkurt I, Ince S, Fidan AF, Ozdemir A (2008) The effects of dietary supplementation of different amount of Yucca schidigera powder (Sarsaponin 30®) on blood and tissue antioxidant defense systems and lipid peroxidation in rats. J Anim Vet Adv 7:1413–1417

[17] Ohkawa H, Ohishi N, Yagi K (1979) Assay for lipid peroxides in animal tissues by thiobarbituric acid reaction. Anal Biochem 95:351–358. 10.1016/0003-2697(79)90738-3 36810 10.1016/0003-2697(79)90738-3

[18] Beutler E, Duron O, Kelly BM (1993) Improved method for the determination of blood glutathione. Journal of Laboratoty and Clinical Medicine 61:882–888 13967893

[19] Sun Y, Oberley LW, Li Y (1988) A simple method for clinical assay of superoxide dismutase. Clin Chem 34:497–500. 10.1093/clinchem/34.3.497 3349599

[20] Aebi H (1974) Catalase in vitro. In: U. Bergmeyer (Ed.), Methods of enzymatic analysis. Academic Press, New York and London, pp 673–677. 10.1016/B978-0-12-091302-2.50032-3

[21] Elsisy RA, El-Magd MA, Abdelkarim MA (2021) High-fructose diet induces earlier and more severe kidney damage than high-fat diet on rats. Egyptian Journal of Histology 44(2):535–544. 10.21608/ejh.2020.31508.1304

[22] Abdel-Kawi SH, Hassanin KM, Hashem KS (2016) The effect of high dietary fructose on the kidney of adult albino rats and the role of curcumin supplementation: a biochemical and histological study. Beni-suef Univ J Basic Appl Sci 5(1):52–60. 10.1016/j.bjbas.2015.12.002

[23] Suvarna CM, Malothu N, & Guntupalli C (2024) Investigation of Lupinus angustifolius extracts against high-fructose diet-induced metabolic syndrome in male Wistar rats. Pharmacognosy Research, 16(2). 10.5530/pres.16.2.49

[24] Mirzaei R, Khosrokhavar R, ArbabiBidgoli S (2023) The role of high-fructose diet in liver function of rodent models: a systematic review of molecular analysis. Iran Biomed J 27(6):326–339 38193285 10.52547/ibj.3965 PMC10826909

[25] Vrbjar N, Vlkovicova J, Snurikova D, Kalocayova B, Zorad S, Culafic T, Tepavčeviç S, Totava L, Radosinska D, Kollarova M, Radosinska J (2023) Alterations in oxidative stress markers and Na, K-ATPase enzyme properties in kidney after fructose intake and quercetin intervention in rats. Life 13(4):931. 10.3390/life13040931 37109460 10.3390/life13040931 PMC10142800

[26] Yang Y, Li J, Wei C, He Y, Cao Y, Zhang Y, He J (2019) Amelioration of nonalcoholic fatty liver disease by swertiamarin in fructose-fed mice. Phytomedicine 59:152782. 10.1016/j.phymed.2018.12.005 31005808 10.1016/j.phymed.2018.12.005

[27] Kar E, Kar F, Can B, ÇakırGündoğdu A, Özbayer C, Koçak FE, Şentürk H (2024) Prophylactic and therapeutic efficacy of boric acid on lipopolysaccharide-induced liver and kidney inflammation in rats. Biol Trace Elem Res 202(8):3701–3713. 10.1007/s12011-023-03941-4 37910263 10.1007/s12011-023-03941-4

[28] Khaliq H, Juming Z, Ke-Mei P (2018) The physiological role of boron on health. Biol Trace Elem Res 186:31–51. 10.1007/s12011-018-1284-3 29546541 10.1007/s12011-018-1284-3

[29] Nielsen FH (2008) Is boron nutritionally relevant? Nutr Rev 66(4):183–191. 10.1111/j.1753-4887.2008.00023.x 18366532 10.1111/j.1753-4887.2008.00023.x

[30] Chetehouna S, Derouiche S, Reggami Y, Boulaares I (2024) Sonchus maritimus Extract-loaded niosomes bioconjugated by linoleic acid in hepatic encephalopathy induced by high-fructose diet in albino Wistar rats. Archives of Razi Institute 79(1):189. 10.32592/ARI.2024.79.1.189 39192951 10.32592/ARI.2024.79.1.189 PMC11345485

[31] Özkan H, Kutlu T (2020) The relationship of fructose consumption with MDA levels in rat liver and its effect on the expression levels of COX-2 and NRF-2 genes. Ankara Üniversitesi Veteriner Fakültesi Dergisi 67(4):387–392. 10.33988/auvfd.645713

[32] Iskender H, Yenice G, Dokumacioglu E, Hayirli A, Sevim C, Dokumacioglu A, TerimKapakin KA (2020) Astaxanthin alleviates renal damage of rats on high fructose diet through modulating NFκB/SIRT1 pathway and mitigating oxidative stress. Arch Physiol Biochem 126(1):89–93. 10.1080/13813455.2018.1493609 30081678 10.1080/13813455.2018.1493609

[33] Yilmaz Demirtas C, Bircan FS, Pasaoglu OT, Turkozkan N (2018) The effects of resveratrol on hepatic oxidative stress in metabolic syndrome model induced by high fructose diet. Bratisl Med J 119:36–40. 10.4149/BLL_2018_008 10.4149/BLL_2018_008 29405729

[34] Kan F, Kucukkurt I (2023) The effects of boron on some biochemical parameters: A review. J Trace Elements Med Biology 127249. 10.1016/j.jtemb.2023.127249 10.1016/j.jtemb.2023.127249 37413926

[35] Çakir S (2024) Effect of Boric Acid on Metabolic Peptides and Some Biochemical Parameters in Experimental Diabetic Rats. Biol Trace Elem Res 202(3):1001–1008. 10.1007/s12011-023-03910-x 37872360 10.1007/s12011-023-03910-x

[36] Demirdogen RE (2020) Relationship among blood boron level, diabetes mellitus, lipid metabolism, bone metabolism and obesity: Can boron be an efficient indicator for metabolic diseases. Health Sci. J 14(1):1–11. 10.36648/1791-809X.14.1.689

[37] Aydın S, Demirci S, Doğan A, Sağraç D, Kaşıkcı E, Şahin F (2019) Boron containing compounds promote the survival and the maintenance of pancreatic β-cells. Mol Biol Rep 46(5):5465–5478. 10.1007/s11033-019-05002-3 31368021 10.1007/s11033-019-05002-3

[38] Basoglu A, Sevinc M, Birdane FM, Boydak M (2002) Efficacy of sodium borate in the prevention of fatty liver in dairy cows. J Vet Intern Med 16(6):732–735. 10.1111/j.1939-1676.2002.tb02416.x 12465773 10.1892/0891-6640(2002)016<0732:eosbit>2.3.co;2

[39] Kuru R, Yilmaz S, Balan G, Tuzuner BA, Tasli PN, Akyuz S, Sahin F (2019) Boron-rich diet may regulate blood lipid profile and prevent obesity: A non-drug and self-controlled clinical trial. J Trace Elem Med Biol 54:191–198. 10.1016/j.jtemb.2019.04.021 31109611 10.1016/j.jtemb.2019.04.021

[40] Ince S, Kucukkurt I, Demirel HH, Arslan-Acaroz D, Varol N (2020) Boron, a trace mineral, alleviates gentamicin-induced nephrotoxicity in rats. Biol Trace Elem Res 195(2):515–524. 10.1007/s12011-019-01875-4 31446563 10.1007/s12011-019-01875-4

[41] Özkan E, Karabağ F (2020) Investigation of boron effect on trace elements and antioxidant capacity in paracetamol-induced nephrotoxicity model. Veteriner Hekimler Derneği Dergisi 91(1):25–35. 10.33188/vetheder.557918

[42] Iskender H, Dokumacioglu E, Saral S, Yenice G, Sevim C (2018) NF-κB, TNF-α and IL-6 levels in liver and kidney of high-fructose-fed rats. J Adv Med Pharmaceutical Sci 18(3):1–7. 10.9734/JAMPS/2018/44823

[43] Botezelli JD, Cambri LT, Ghezzi AC, Dalia RA, Voltarelli FA, de Mello MAR (2012) Fructose-rich diet leads to reduced aerobic capacity and to liver injury in rats. Lipids Health Dis 11:1–9. 10.1186/1476-511X-11-78 22713601 10.1186/1476-511X-11-78 PMC3473252

[44] Yuan L, Han X, Li W, Ren D, Yang X (2016) Isoorientin prevents hyperlipidemia and liver injury by regulating lipid metabolism, antioxidant capability, and inflammatory cytokine release in high-fructose-fed mice. J Agric Food Chem 64(13):2682–2689. 10.1021/acs.jafc.6b00290 26961674 10.1021/acs.jafc.6b00290

[45] Masterjohn C, Park Y, Lee J, Noh SK, Koo SI, Bruno RS (2013) Dietary fructose feeding increases adipose methylglyoxal accumulation in rats in association with low expression and activity of glyoxalase-2. Nutrients 5(8):3311–3328. 10.3390/nu5083311 23966111 10.3390/nu5083311 PMC3775256

[46] Mengesha T, Gnanasekaran N, Mehare T (2021) Hepatoprotective effect of silymarin on fructose induced nonalcoholic fatty liver disease in male albino Wistar rats. BMC Complementary Med Therapies 21:1–13. 10.1186/s12906-021-03275-5 10.1186/s12906-021-03275-5 PMC8011178 33785007

[47] Wang C, Shi Y, Gu W, Wang C, Xu Y, Li L, Zhang S, Zhi H, Ruan H, Kong J, Duan L, Tang S (2023) Protective role of boron on hepatotoxicity and oxidative stress induced by trichloroacetic acid. Environmental Sciences Europe 35(1):74. 10.1186/s12302-023-00775-8

[48] Ahangarpour A, Mohammadian M, Dianat M (2012) Antidiabetic effect of hydroalcholic Urtica dioica leaf extract in male rats with fructose-induced insulin resistance. Iranian journal of medical sciences 37(3):181 23115450 PMC3470082

[49] Zaki SM, Fattah SA, Hassan DS (2019) The differential effects of high-fat and high–fructose diets on the liver of male albino rat and the proposed underlying mechanisms. Folia Morphol 78(1):124–136. 10.5603/FM.a2018.0063 10.5603/FM.a2018.0063 30009361

[50] Aşcı H, Saygın M, Yeşilot Ş, Topsakal Ş, Cankara FN, Özmen Ö, Savran M (2016) Protective effects of aspirin and vitamin C against corn syrup consumption-induced cardiac damage through sirtuin-1and HIF-1α pathway. Anatolian Journal of Cardiology 16(9). 10.5152/AnatolJCardiol.2015.6418 10.5152/AnatolJCardiol.2015.6418 PMC5331347 26645266

[51] Lechner M, Lirk P, Rieder J (2005) Inducible nitric oxide synthase (iNOS) in tumor biology: the two sides of the same coin. In Seminars in cancer biology 15 (4):277–289. Academic Press 10.1016/j.semcancer.2005.04.004 10.1016/j.semcancer.2005.04.004 15914026

[52] Youssef EA (2016) Effect of metformin on histopathological and immunohistochemical changes induced by high fructose intake in liver and brain of rats. J Biosci Appl Res 2(1):64–80. 10.21608/jbaar.2015.106489

